# Results of PCI with Drug-Eluting Stents in an All-Comer Population Depending on Vessel Diameter

**DOI:** 10.3390/jcm9020524

**Published:** 2020-02-14

**Authors:** Janusz Dola, Beata Morawiec, Wojciech Wańha, Ewa Nowalany-Kozielska, Wojciech Wojakowski, Damian Kawecki

**Affiliations:** 1 2nd Department of Cardiology, School of Medicine with the Division of Dentistry in Zabrze, Medical University of Silesia, 41-800 Zabrze, Poland; alodnet@tlen.pl (J.D.); beamorawiec@wp.pl (B.M.); ewakozielska@wp.pl (E.N.-K.); 2 3rd Division of Cardiology, Medical University of Silesia, 40-635 Katowice, Poland; wojciechwanha@gmail.com (W.W.);

**Keywords:** PCI, DES, vessel size, MACCE

## Abstract

Long-term outcome after percutaneous coronary intervention (PCI) depends on vessel diameter; however, there is insufficient evidence on particular drug-eluting stent (DES) types in this setting. The aim of the study was to assess long-term performance of PCI depending on stented vessel size and DES generations. This observational study from a prospective Registry of PCI with DES assessed safety (stent thrombosis) and efficacy (major adverse cardiac and cerebrovascular event (MACCE)) of the implantation of first- (DES1) or second-generation DESs (DES2) in small and large vessels. Of 699 patients included in the analysis, 337 (48%) patients underwent PCI in small vessels. PCI in small vessels, especially the left anterior descending artery (LAD) (hazard ratio (HR) 2.6, 95% confidence interval (CI) 1.5–4.5), was associated with a higher rate of MACCEs than that in large vessels (20% vs. 14%, *p* = 0.025) with no difference in the rate of stent thrombosis (ST). No significant difference in safety and efficacy was found between DES1 and DES2 in small vessels. For large vessels, a higher incidence of MACCEs (21% vs. 9.2%, *p* = 0.002) driven by a higher rate of re-PCI (15% vs. 6%, *p* = 0.006) and a higher rate of cumulative stent thrombosis (3.5% vs. 0.5%, *p* = 0.04) was shown for DES1 than DES2. In multivariate analysis, DES1 was a significant risk factor for MACCEs in large, but not in small vessels. The risk of PCI in small vessels, especially LAD, remains high independent of the type of DES. In contrast, DES2 as a modifiable variable during PCI of a large lesion might improve long-term prognosis.

## 1. Introduction

Percutaneous coronary intervention (PCI) revolutionized the treatment of coronary artery disease, while the use of drug-eluting stents (DES) has become a well-established and widely available therapeutic method. 

Despite the early reports on reduced rate of restenosis and repeat revascularization in comparison to bare metal stents (BMS) [1,2,3,4], some meta-analyses linked the use of DES to a higher risk for long-term mortality secondary to the stent thrombosis (ST) [5,6,7,8,9].

Thinner struts and biodegradable polymers of newer-generation DESs resulted in up to 50% reduction in stent thrombosis when compared to early generation DESs [10,11,12,13]. Several reports showed that not only the type of stent but also the diameter of the vessel undergoing PCI is crucial for the development of major adverse cardiac and cerebrovascular events (MACCEs) with higher risk of events after implantation of a larger DES (more than 3 mm) [14,15,16]. On the contrary, the advantage of DES over BMS for both safety and efficacy was reported for small vessels [17,18,19]. 

To date, there is insufficient evidence on the performance of particular DES types depending on vessel diameter.

The aim of the study was to assess the long-term safety and efficacy of first- (DES1) and second-generation DESs (DES2) depending on the size of the vessel.

## 2. Experimental Section

### 2.1. Study Design

The study was a sub-analysis from an all-comer retrospective Katowice–Zabrze Registry of patients treated with PCI with the implantation of DES. The analysis included patients after PCI with DES for coronary artery disease (CAD) or acute coronary syndrome (ACS) admitted to the Second Department of Cardiology, Zabrze, Medical University of Silesia, Katowice, Poland between 1 January, 2001 and 31 December, 2014.

**Figure 1:**
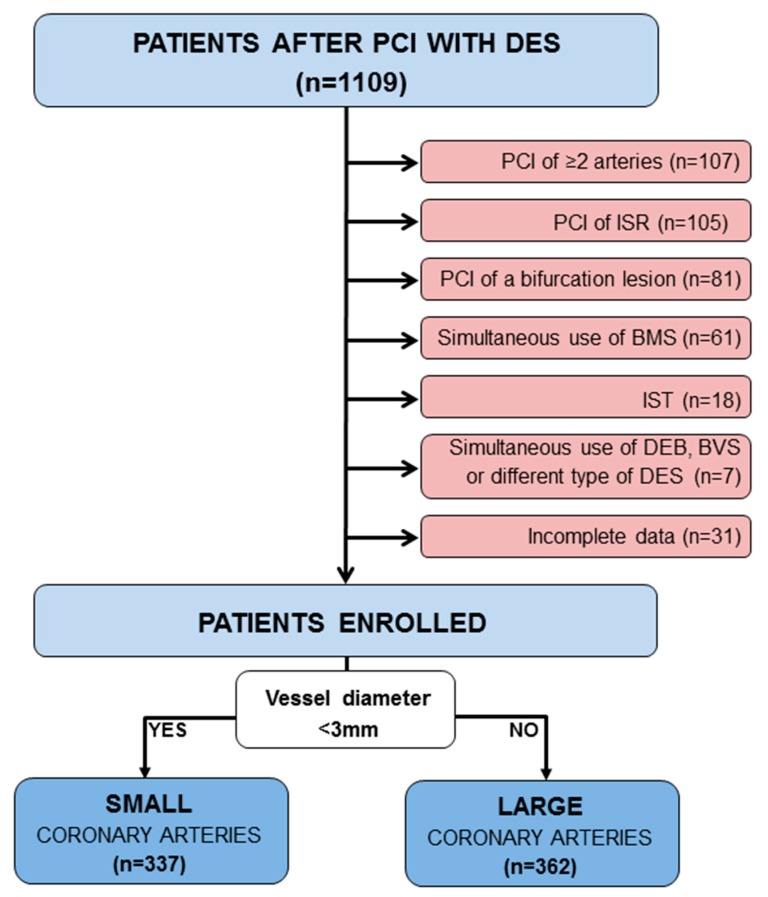
Study chart. ISR—in stent restenosis, IST—in stent thrombosis, BMS—bare metal stents, BVS—bioresorbable vascular scaffold, DEB—drug-eluting balloon, DES—drug-eluting stent, PCI—percutaneous coronary intervention.

Exclusion criteria were simultaneous PCI of two or more coronary arteries, use of BMS/bioresorbable vascular scaffold (BVS)/drug-eluting balloon (DEB) or two different types of DESs in the same procedure, PCI of coronary bifurcation, in-stent restenosis or thrombosis, incomplete data (Figure 1).

The study population was divided by vessel diameter, assessed by the diameter of implanted DES, into groups of small and large coronary arteries, considering 3 mm as the discriminant diameter. Secondly, the study population was divided depending on the DES used into first- (DES1) and second-generation (DES2) DES groups.

The study was approved by the local ethical committee of Medical University of Silesia (decision no. KNW/0022/KB/59/11). Basic anthropometric, clinical, angiographic, and procedural data were collected retrospectively for each patient based on available medical records. Clinical features included age, sex, body mass index, CAD risk factors (smoking, hypertension, hyperlipidemia, diabetes mellitus, obesity, positive family history), past medical history, co-morbidities, left ventricular ejection fraction, and discharge diagnosis. Procedural and angiographic characteristics included location of the lesion, severity of stenosis, the American College of Cardiology/American Heart Association (ACC/AHA) lesion type, thrombus, calcifications, number, length, and diameter of DES per lesion. Stents were chosen according to the operator’s decision according to current practice, knowledge, and individual experience and preferences regarding particular stent characteristics suitable to type of the lesion. Dual antiplatelet therapy (acetylsalicylic acid and clopidogrel) was prescribed for up to 12 months after the procedure in each patient. Baseline clinical, angiographic, and procedure-related data were retrospectively collected from medical records. 

### 2.2. Coronary Stenting

Paxel, LUC-Chopin, LUC-Chopin2, Carlo, Prolim (Balton, Warsaw, Poland), Partner (Lepu Medical Technology Co., Beijing, China), and Taxcor (Opto Eurocor Healthcare Ltd., Bengaluru, India) were classified as first-generation DESs.

Resolute Integrity (Medtronic, Minneapolis, MN, USA), Xience Prime (Abbott Vascular, Santa Clara, CA, USA), Promus Element (Boston Scientific, Natick, MA, USA), Orsiro (Biotronik AG, Bülach, Switzerlandand), CRE8 (Alvimedica, Istanbul, Turkey), Genous (OrbusNeich Medical Technologies, Fort Lauderdale, FL, USA), and Alex (Balton, Warsaw, Poland) were classified as second-generation DESs.

### 2.3. Follow-Up

Long-term follow-up was carried out for all patients and was closed one year after inclusion of the last patient or with the occurrence of an endpoint. All information was obtained from medical records of the enrolling center. If no data were available, information on clinical endpoints was obtained from National Health Care System.

The primary efficacy endpoint was defined as a composite of major adverse cardiac and cerebrovascular events (MACCEs) and included all-cause death, non-fatal myocardial infarction (MI), target-vessel revascularization (TVR), and stroke. The secondary endpoints were individual components of the primary endpoint: all-cause death, MI, TVR, stroke.

The primary safety endpoint was definite stent thrombosis, defined according to the Academic Research Consortium’s definition. 

### 2.4. Statistics

Variables were checked for normality of distribution with Shapiro–Wilks test. Continuous variables are presented as mean and standard deviation (SD) or median and 25th and 75th percentile and were compared with Student T test or Mann–Whitney test. Categorical variables are presented as percentages and were compared with chi-square test or Fisher’s test for the groups <5. The Kaplan–Meier method was used to present estimated incidence of endpoints and the long-rank test was used to assess differences between groups. Clinical, hemodynamic, and procedural characteristics that differed significantly between groups were used for univariate Cox regression for assessing the influence on clinical endpoints. Variables significant in univariate analysis were the substrate for the multivariable model. All tests were two-tailed and the *p* value of <0.05 was considered significant. Statistical analysis was performed with GraphPad QuickCalcs (La Jolla, CA, USA), MedCalc 18.2.1 (MedCalc Software, Ostend, Belgium), and IBM SPSS Statistics 22 (IBM, Armonk, New York, NY, USA). 

## 3. Results

### 3.1. Study Population

**Figure 2:**
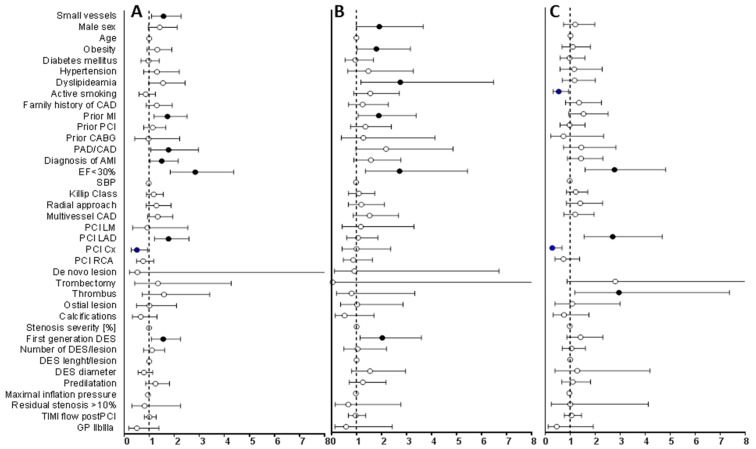
Univariate Cox proportional hazard model for long-term major adverse cardiac and cerebrovascular events (MACCEs) in all patients (**A**), large vessels (**B**), small vessels (**C**). Data are presented as HR and 95% CI. Full dots represent HR of statistically significant risk factors, empty dots represent HR of not statistically significant variables, bars represent 95% CI, dotted line shows HR = 1. CAD—coronary artery disease, MI—myocardial infarction, PCI—percutaneous coronary intervention, CABG—coronary artery bypass grafting, PAD/CAD—peripheral and carotid artery disease, SBP—systolic blood pressure, LM—left main artery, LAD—left anterior descending artery, Cx—circumflex artery, RCA—right coronary artery, DES—drug-eluting stent, TIMI—thrombolysis in myocardial infarction, GP—glycoprotein, HR—hazard ratio, CI—confidence interval.

From the total of 1109 patients undergoing PCI with DES between 2011 and 2014, 410 patients were excluded (Figure 2). The remaining 699 patients, who entered the analysis, were divided into a group of small coronary arteries (337 patients) and a group of large coronary arteries (362 patients) (Figure 2). Patients were further stratified into groups depending on the type of DES implanted. In the group of small coronary arteries, DES1 was implanted in 104 patients (31%), DES2 in 233 patients (69%). In the group of large coronary arteries, DES1 was implanted in 144 patients (40%), DES2 in 218 patients (60%).

### 3.2. Clinical Characteristics

Both main groups had similar clinical profiles, with more obese patients in the large vessels group (Table 1). 

In the group of small coronary arteries, patients after implantation of DES1 and DES2 had a similar clinical profile, with more patients with reduced ejection fraction (≤30%) (37 patients, 16% vs. 8 patients, 7.7%; *p* = 0.01) and the history of coronary artery bypass grafting (CABG) (19 patients, 8.1% vs. 1 patient, 1.0%; *p* = 0.04) in DES2. There was also no major clinical difference between DES1 and DES2 in the group of large coronary arteries.

### 3.3. Procedural Characteristics

Angiographic and procedural characteristics of the studied population are presented in Table 2. The most frequently stented artery in the general population was the left anterior descending artery (LAD), symmetrically distributed between groups of small and large vessels, PCI of left main artery (LM) was not represented in the small vessels group. No difference between PCI of LAD in small and large vessels was accompanied with more PCI of circumflex artery (Cx) in small vessels, and right coronary artery (RCA) in large vessels. The PCI in small vessels was characterized by more severe stenosis of treated lesion (*p* = 0.005), more frequent PCI with more than one stent (*p* < 0.001), and a higher rate of predilatation (*p* < 0.001). DES2 was implanted in 233 patients (69%) in the group of small coronary arteries and in 218 patients (60%) in the group of large coronary arteries (*p* = 0.014). 

Angiographic and procedural characteristics were similar between DES1 and DES2 in both groups. Considering the group of small coronary arteries only, multivessel coronary disease was more frequent in DES2 (128 patients, 55% vs. 43, 41%; *p* = 0.02).

### 3.4. Follow-Up

Median follow-up was 878 (515;1278) days. Patients after PCI in small vessels experienced more MACCEs than patients with PCI in large vessels (20% vs. 14%, *p* = 0.025); however, no statistically significant difference was achieved for particular components of MACCEs. PCI was equally safe in both cases, with no statistically significant difference in stent thrombosis (0.9% in small vs. 1.7% in large vessels, *p* = 0.51).

In the group of small vessels, both types of DESs were equally safe and effective, with a trend toward more MACCEs in DES1 than in DES2 (26% vs. 18%, respectively, *p* = 0.08). The rate of MACCEs was the highest after PCI in LAD (27%) when compared to PCI in Cx (7.8%) and PCI in RCA (16%), *p* = 0.009.

A significantly higher rate of MACCEs was observed for DES1 than for DES 2 implanted in large vessels (21% vs. 9.2%, respectively, *p* = 0.002), driven by a higher incidence of re-PCI (15% vs. 6%, *p* = 0.006) and a tendency toward higher incidence of acute myocardial infarction (AMI) and stroke (*p* = 0.08 for both endpoints). DES2 were also safer in large vessels than DES1 with a lower rate of cumulative stent thrombosis (0.5% vs. 3.5%, *p* = 0.04). Long-term follow-up data are summarized in Table 3.

The influence of baseline clinical and procedural variables on the occurrence of MACCEs was assessed with a Cox proportional hazard model. 

In univariate analysis, small size of the vessel undergoing PCI was a significant risk factor of MACCEs in the total population (hazard ratio (HR) 1.58, 95% confidence interval (CI) 1.10–2.28, *p* = 0.014). Further parameters significantly increasing the risk of MACCE for the total population were the implantation of DES1 (HR 1.57, 95% CI 1.09–2.26, *p* = 0.014), PCI of LAD (HR 1.78, 95% CI 1.22–2.6, *p* = 0.003), prior AMI (HR 1.74, 95% CI 1.2–2.52, *p* = 0.004), PAD/CAD (HR 1.78, 95% CI 1.07–2.98, *p* = 0.03), PCI in AMI (HR 1.51, 95% CI 1.05–2.17, *p* = 0.026), and low ejection fraction (HR 2.85, 95% CI 1.85–4.38, *p* < 0.001) (Figure 2). 

In the group of small vessels, significant risk factors for MACCEs were low ejection fraction (HR 2.78, 95% CI 1.6–4.83, *p* < 0.001), PCI of LAD (HR 2.71, 95% CI 1.56–4.69, *p* < 0.001), and thrombus (HR 2.96, 95% CI 1.19–7.37, *p* = 0.02); the risk of MACCE was increased in large vessels with the implantation of DES1 (HR 2.04, 95% CI 1.15–3.6, *p* = 0.014), in male (HR 1.92, 95% CI 1.0–3.67, *p* = 0.05) and obese patients (HR 1.8, 95% CI 1.03–3.16, *p* = 0.04), with prior AMI (HR 1.9, 95% CI 1.07–3.39, *p* = 0.03), dyslipidemia (HR 2.76, 95% CI 1.18–6.48, *p* = 0.02), and low ejection fraction (HR 2.73, 95%CI 1.36–5.45, *p* = 0.005) (Figure 2).

Considering a tendency toward higher rate of death and stroke in the small vessels group, an additional Cox hazard model was calculated to assess potential influence of the extent of CAD or the procedure on the outcome. Neither the extent of CAD nor the length/number of stents per lesion reached statistical significance in this analysis, with a tendency for the number of DESs per lesion (*p* = 0.75 for death and *p* = 0.59 for stroke).

**Figure 3:**
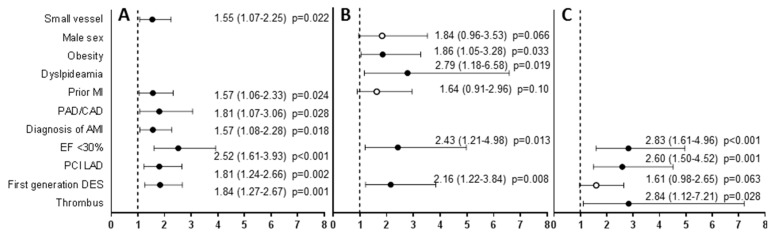
Multivariate Cox proportional hazard model for long-term MACCE in all patients (**A**), large vessels (**B**), small vessels (**C**). Data are presented as HR (95% CI). Full dots represent hazard ratio of statistically significant risk factors, empty dots represent hazard ratio of not statistically significant variables, bars represent 95% CI, dotted line shows HR = 1. MI—myocardial infarction, PAD/CAD—peripheral/carotid artery disease, EF—ejection fraction, PCI—percutaneous coronary intervention, LAD—left anterior descending artery, DES—drug-eluting stent, HR—hazard ratio, CI—confidence interval.

All the risk factors of MACCEs for the total population and small vessels group remained significant in multivariate Cox analysis. In the group of large vessels, only DES1, low ejection fraction, obesity, and dyslipidemia remained significant risk factors for MACCEs (Figure 3).

**Figure 4:**
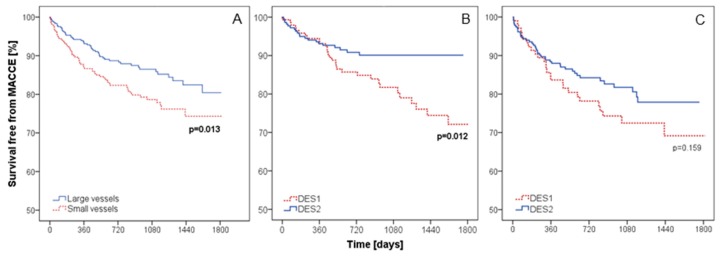
Kaplan–Meier curves for long-term survival free from MACCEs in all patients depending on the size of the vessel (**A**), large vessels group (**B**), and small vessels group (**C**) depending on the type of DES. MACCE—major adverse cardiac and cerebrovascular events, DES—drug-eluting stent.

According to Kaplan–Meier analysis, the long-term survival free from MACCEs in the small vessels group was lower than that in the large vessels group (log-rank *p* = 0.013) (Figure 4A).

In the subgroup of small vessels, the type of DES was not a discriminant of MACCE-free survival (log-rank *p* = 0.159, Figure 4B). Contrarily, in the subgroup of large vessels, patients after implantation of DES2 had significantly better MACCE-free survival than after the implantation of DES1 (log-rank *p* = 0.012, Figure 4C).

## 4. Discussion

Based on the results of this study comparing groups of patients requiring percutaneous coronary intervention in small (<3 mm) or large (≥3 mm) coronary arteries with the use of two generations of drug-eluting stents, we report five major findings.

First, the risk of long-term MACCEs is higher after PCI of a small than a large coronary artery, which is mainly driven by the need for repeat revascularization. This well-known fact has already been reported several times in the literature, explaining worse outcome of PCI in small vessels by clinical characteristics correlated with such anatomy and specific device utilization [20,21]. In our population, no major differences in clinical and procedural characteristics were reported between large and small vessels, however further observations listed below put some new, specific insights into this rule.

Accordingly, considering PCI of small coronary arteries, the procedure in LAD was a strong risk factor for adverse events compared to PCI of any other coronary artery. Prognosis of PCI of large coronary arteries was independent of the stented artery. In other words, PCI of a small LAD increased the risk of long-term MACCEs almost three-fold than any other coronary artery, which was not reported for large LAD. Similar observation was reported previously for LAD to be one of the predictors for stent thrombosis [22,23]. We show that the risk of MACCEs of PCI in LAD is higher than that in other vessel only if the diameter is lower than 3 mm. This effect of LAD as a single risk factor was not shown for large vessels. This observation merits further investigation.

Third, the type of DES is not a discriminant of long-term MACCEs in small arteries, as neither DES1 nor DES2 turned out as a risk factor in this group. Contrarily, PCI in large coronary arteries was more risky regarding long-term events when DES1 was implanted. Higher rates of clinical and angiographic restenosis in small vessels, and thus pathology leading to repeated revascularization, was described previously in large registries for both older and newer generations of DESs [24,25,26]. Older DESs were associated with higher rate of stent thrombosis in small than in large arteries. However, such analysis seems of less clinical value, as the size of the vessel undergoes no modifications while facing PCI of a lesion in a particular patient. While dealing with a lesion in a small vessel, the type of stent is of less influence on the prognosis than the size of the vessel itself. In contrast, DES2 should be preferred over DES1 during PCI whenever possible in large arteries due to a lower risk of MACCEs with the newer generation of DES in this group.

Fourth, in the group of small coronary arteries, further decrease in vessel diameter below 3 mm did not influence prognosis of PCI. Increasing diameter of the vessel over 3 mm in large coronary arteries also did not change the prognosis. This observation, confirming previous findings [27], leads to the conclusion that one specific cutoff for small and large vessels is sufficient for risk stratification and decision on optimal management of particular lesion.

Fifth, the presence of a thrombus in the lesion undergoing PCI was a strong risk factor for adverse events in small coronary arteries, but thrombectomy was not identified as a protective factor. Facing an intensive debate on the current role of thrombectomy in PCI worldwide [28,29,30], this study adds little evidence as it was not the aim of the analysis to assess the role of thrombectomy. Nevertheless, this additional observation would state against routine use of this method during PCI. 

Lastly, long-term prognosis after stenting of small coronary arteries seems to be dependent more on angiographic and procedural factors (PCI of LAD, thrombus), in contrast to stenting of large coronary arteries, where clinical characteristics were balanced with procedural parameters (obesity, dyslipidemia, DES1). Considering the clinical setting, one should focus treatment strategy on modifiable factors in order to maximally decrease the risk of MACCEs.

Based on the findings listed above, following high-risk models of patients might be defined.

First, considering angiographic findings in all patients referred for coronary angiogram, one should be aware of the highest risk of PCI with DES1 for any coronary lesion below 3 mm of diameter, especially LAD, in a patient admitted for AMI, with severe systolic dysfunction of the left ventricle and the history of myocardial infarction, peripheral, or carotid artery disease.

Second, in case the diameter of the lesion planned for PCI is lower than 3 mm, the procedure should be strongly analyzed when dealing with LAD, especially in patients with low ejection fraction. Thrombectomy might not influence the outcome if the thrombus is found on coronary angiogram.

Third, for patients in whom significant lesion is found in a coronary artery of 3 mm or larger, the procedure with the use of DES1 should be avoided. Post-PCI management should include closer monitoring of obese patients with dyslipidemia and low ejection fraction.

Some limitations have to be mentioned. DESs within the same generation were not directly compared to each other, the heterogeneity of efficacy and safety for different DESs within each generation may confound study outcomes to a certain extent. Due to limited patient population available for analysis, the number of safety endpoints is respectively lower and therefore limits proper analysis. The cutoff of 3 mm for vessel size was arbitrary but adopted from previous analysis [14] and consistent with median vessel size in the studied population, thus enabling relatively appropriate distribution of patients in study subgroups.

## 5. Conclusions

In conclusion, the study proves the high risk of PCI for small vessels, identifies the multifactorial high-risk patient profile, and indicates the type of DES as a modifiable variable in the decision-making process during PCI of a lesion with predefined clinical and procedural set of characteristics in an individual patient.

## Figures and Tables

**Table 1:** Clinical characteristics.

	All Patients (*n* = 699)	Large Vessels (*n* = 362)	Small Vessels (*n* = 337)	*p* Value ^1^
Male sex	437 (63%)	232 (64%)	205 (61%)	0.39
Age [years]	65 (58;72)	65 (58;72)	64 (58;72)	0.95
**Risk Factors for coronary artery disease**				
Obesity	281 (40%)	159 (44%)	122 (36%)	0.045
Hypertension	574 (82%)	293 (81%)	281 (83%)	0.43
Diabetes mellitus	298 (43%)	153 (42%)	145 (43%)	0.88
Dyslipidemia	503 (72%)	265 (73%)	238 (71%)	0.45
Smoking	292 (42%)	160 (44%)	132 (39%)	0.19
Familial History of CAD	189 (27%)	97 (27%)	92 (27%)	0.93
**Comorbidities**				
Coronary artery disease	16 (2.3%)	6 (1.7%)	10 (3.0%)	0.31
Peripheral artery disease	55 (7.9%)	22 (6.1%)	33 (9.8%)	0.09
Chronic renal disease	88 (13%)	44 (12%)	44 (13%)	0.73
Prior AMI	180 (26%)	82 (23%)	98 (29%)	0.06
Prior PCI	239 (34%)	117 (32%)	122 (36%)	0.30
Prior CABG	35 (5.0%)	15 (4.1%)	20 (5.9%)	0.30
**Diagnosis**				
STEMI	49 (7.0)	26 (7.2%)	23 (6.8%)	0.88
NSTEMI	189 (27%)	97 (27%)	92 (27%)	0.93
Unstable angina	165 (24%)	93 (26%)	72 (21%)	0.18
LVEF ≤30%	80 (11%)	35 (9.7%)	45 (13%)	0.15

Data presented as n (%) or median (25th; 75th percentile). CAD—coronary artery disease, AMI—acute myocardial infarction, PCI—percutaneous coronary intervention, CABG—coronary artery bypass grafting, STEMI—stent thrombosis-segment elevation myocardial infarction, NSTEMI—non-stent thrombosis-segment elevation myocardial infarction, LVEF—left ventricle ejection faction. ^1^ Between groups of large and small vessels.

**Table 2:** Angiographic and procedural characteristics.

	All Patients (*n* = 699)	Large Vessels (*n* = 362)	Small Vessels (*n* = 337)	*p* Value ^1^
**Angiographic characteristics**				
ACC/AHA lesion B2-C	377 (55)	199 (55)	178 (53)	0.86
Stenosis severity [%]	80 (70;95)	80 (70;95)	90 (70;95)	0.005
Significant calcification	75 (11)	38 (11)	37 (11)	0.90
Ostial lesion	46 (6.6)	27 (7.5)	19 (5.6)	0.36
Thrombus	30 (4.3)	18 (5.0)	12 (3.6)	0.46
Multivessel disease	345 (49)	174 (48)	171 (51)	0.50
**Procedural characteristics**				
DES 2	451 (65)	218 (60)	233 (69)	0.014
Stented artery				
LM	22 (3.1)	22 (6.1)	0 (0)	<0.001
LAD	331 (47)	180 (50)	151 (45)	0.20
Cx	124 (18)	47 (13)	77 (23)	0.001
RCA	183 (26)	109 (30)	74 (22)	0.02
Bypass	2 (0.3)	2 (0.6)	0 (0)	0.50
Number of DESs per lesion				<0.001
1	576 (82)	316 (87)	260 (77)	
2	106 (15)	41 (11)	65 (19)	
3	14 (2.0)	5 (1.4)	9 (2.7)	
4	3 (0.4)	0 (0)	3 (0.9)	
DES length per lesion [mm]	22 (15;29)	22 (15;29)	22 (15;30)	0.036
DES diameter [mm]	3.0 (2.5;3.25)	3.25 (3.0;3.5)	2.5 (2.25;2.75)	<0.001
Primary PCI	402 (58)	212 (59)	190 (56)	0.59
Predilatation	376 (54)	164 (45)	212 (63)	<0.001
Dilatation pressure [atm]	12 (12;16)	14 (12;16)	12 (12;14)	0.001
TIMI 3 flow post PCI	632 (90)	329 (91)	303 (90)	0.70
GPIIb/IIIa inhibitors	47 (6.7)	27 (7.5)	20 (5.9)	0.45
Thrombectomy	15 (2.1)	8 (2.2)	7 (2.1)	1.0
Residual stenosis >10%	20 (2.9)	10 (2.8)	10 (3.0)	1.0

Data presented as n (%) or median (25th; 75th percentile). ACC/AHA—American College of Cardiology/American Heart Association, LM—left main artery, LAD—left anterior descending artery, Cx—circumflex artery, RCA—right coronary artery, SVG—saphenous graft; DES—drug-eluting stent, DES2—second-generation DES, PCI—percutaneous coronary intervention, TIMI—thrombolysis in myocardial infarction, GP—glycoprotein. ^1^ Between groups of large and small vessels.

**Table 3:** Long-term follow-up.

	Total Population, *n* = 699			Large Vessels, *n* = 362			Small Vessels, *n* = 337		
	Small Vessels *n* = 337	Large Vessels *n* = 362	*p* Value	DES 1 *n* = 144	DES 2 *n* = 218	*p* Value	DES 1 *n* = 104	DES 2 *n* = 233	*p* Value
Efficacy									
MACCE	68 (20%)	50 (14%)	0.025	30 (21%)	20 (9.2%)	0.002	27 (26%)	41 (18%)	0.08
Death	13 (3.9%)	6 (1.7%)	0.074	3 (2.1%)	3 (1.4%)	0.67	7 (6.7%)	6 (2.6%)	0.07
Non-fatal AMI	31 (9.2%)	29 (8.0%)	0.58	16 (11%)	13 (6.0%)	0.08	12 (12%)	19 (8.2%)	0.32
Re-PCI (TVR)	34 (10%)	34 (9.4%)	0.76	21 (15%)	13 (6.0%)	0.006	13 (13%)	21 (9.0%)	0.33
Stroke	12 (3.6%)	5 (1.4%)	0.062	4 (2.8%)	1 (0.5%)	0.08	4 (3.8%)	8 (3.4%)	1.00
Safety									
Stent thrombosis ^1^	3 (0.9%)	6 (1.7%)	0.51	5 (3.5%)	1 (0.5%)	0.04	2 (1.9%)	1 (0.4%)	0.23
acute	1 (0.3%)	3 (0.8%)	0.63	3 (2.1%)	0 (0%)	0.06	1 (1%)	0 (0%)	0.31
subacute	1 (0.3%)	2 (0.6%)	1.0	1 (0.7%)	1 (0.5%)	1.00	0 (0%)	1 (0.4%)	1.00
late	1 (0.3%)	1 (0.3%)	1.0	1 (0.7%)	0 (0%)	0.39	1 (0.96%)	0 (0%)	0.31
Very late	0 (0%)	0 (0%)	-	0 (0%)	0 (0%)	-	0 (0%)	0 (0%)	-

Data are presented as n (%). DES—drug-eluting stent, MACCE—major adverse cardiac and cerebrovascular event, AMI—acute myocardial infarction, PCI—percutaneous coronary intervention, TVR—target vessel revascularization. ^1^ Definite stent thrombosis according to ARC (Academic Research Consortium).
